# Evaluation of a Stable Isotope-Based Direct Quantification Method for Dicamba Analysis from Air and Water Using Single-Quadrupole LC–MS

**DOI:** 10.3390/molecules25163649

**Published:** 2020-08-11

**Authors:** Manoj Ghaste, Nicholas C. Hayden, Matthew J. Osterholt, Julie Young, Bryan Young, Joshua R. Widhalm

**Affiliations:** 1 Department of Horticulture and Landscape Architecture, Purdue University, West Lafayette, IN 47907, USA; mghaste@purdue.edu; 2 Center for Plant Biology, Purdue University, West Lafayette, IN 47907, USA; 3 Department of Botany and Plant Pathology, Purdue University, West Lafayette, IN 47907, USA; hayden34@purdue.edu (N.C.H.); mosterho@purdue.edu (M.J.O.); young294@purdue.edu (J.Y.); BryanYoung@purdue.edu (B.Y.)

**Keywords:** dicamba, LC–MS, herbicide volatility, internal standard quantification, selected ion monitoring

## Abstract

Dicamba is a moderately volatile herbicide used for post-emergent control of broadleaf weeds in corn, soybean, and a number of other crops. With increased use of dicamba due to the release of dicamba-resistant cotton and soybean varieties, growing controversy over the effects of spray drift and volatilization on non-target crops has increased the need for quantifying dicamba collected from water and air sampling. Therefore, this study was designed to evaluate stable isotope-based direct quantification of dicamba from air and water samples using single-quadrupole liquid chromatography–mass spectrometry (LC–MS). The sample preparation protocols developed in this study utilize a simple solid-phase extraction (SPE) protocol for water samples and a single-step concentration protocol for air samples. The LC–MS detection method achieves sensitive detection of dicamba based on selected ion monitoring (SIM) of precursor and fragment ions and relies on the use of an isotopically labeled internal standard (IS) (D_3_-dicamba), which allows for calculating recoveries and quantification using a relative response factor (RRF). Analyte recoveries of 106–128% from water and 88–124% from air were attained, with limits of detection (LODs) of 0.1 ng mL^−1^ and 1 ng mL^−1^, respectively. The LC–MS detection method does not require sample pretreatment such as ion-pairing or derivatization to achieve sensitivity. Moreover, this study reveals matrix effects associated with sorbent resin used in air sample collection and demonstrates how the use of an isotopically labeled IS with RRF-based analysis can account for ion suppression. The LC–MS method is easily transferrable and offers a robust alternative to methods relying on more expensive tandem LC–MS/MS-based options.

## 1. Introduction

**Figure 1:**
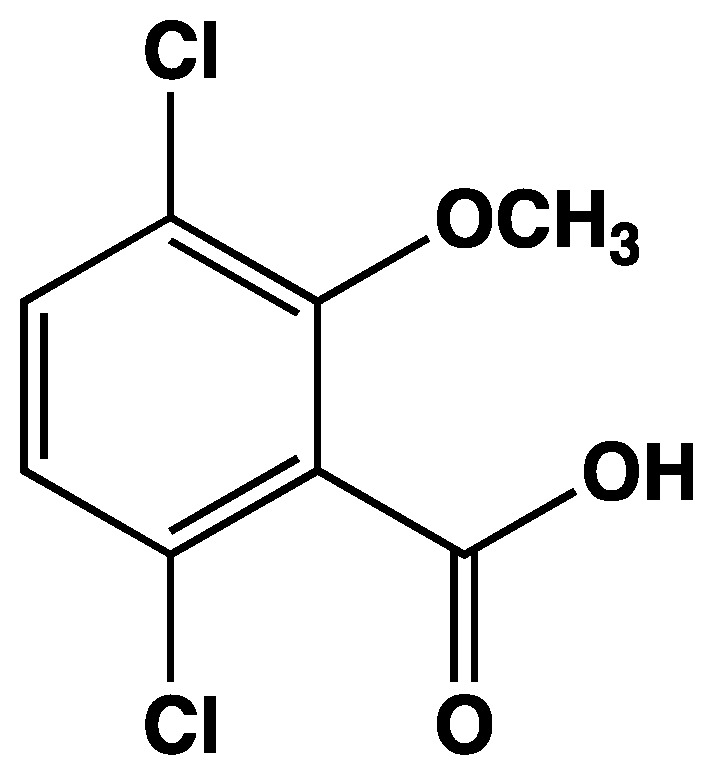
Chemical structure of dicamba.

Dicamba (3,6-dichloro-2-methoxy benzoic acid; Figure 1), a chlorinated derivative of *o*-anisic acid, is an herbicide commonly used to control broadleaf weeds in various sites. Dicamba was originally introduced in the late 1960s and, currently, is commercially sold in its salt-based formulations under brand names such as Banvel^®^, Diablo^®^, Oracle^®^, Vanquish^®^, and XtendiMax^®^ in the United States. Dicamba has a similar mode of action as phenoxy carboxylic acids, which at low doses show similar effects as the class of plant growth hormone auxins and at high concentrations stimulates abnormal cell growth in meristematic cells that results in the blockage of phloem vascular tissue [1]. Off-target effects from herbicides on adjacent fields is a common occurrence in modern agriculture. Dicamba is particularly problematic because, in its acid form, it is moderately volatile and is prone to vapor drift. Dicamba volatility has been widely reported through several on-field and controlled environment studies [2,3,4,5,6,7,8]. Reports have indicated that different crop species such as cotton, groundnut, tomatoes, soybean, watermelon, grapes, ornamentals, and certain tree species in neighboring fields had been damaged by dicamba vapor or drift over the last decade [3,9]. Therefore, there has been demand for rapid, efficient, robust, and sensitive analytical methods to monitor for the presence of dicamba in air and water samples.

Liquid chromatography coupled to mass spectrometry (LC–MS)-based quantitative analytical methods are commonly used to monitor pesticide contamination in environmental and food samples [10,11]. These analytical methods generally require extensive method validation to comply with different regulatory guidelines, which may vary for samples of different origins. Due to its potential to volatilize, accurate quantification of dicamba is not trivial and can be affected by both sample preparation and the detection method used. Most reported methods for monitoring dicamba in water samples use liquid chromatography–tandem mass spectrometry (LC–MS/MS) [12,13,14], whereas others use gas chromatography (GC)–MS in combination with different analyte derivatization procedures [15,16]. Sub-picogram level detection of dicamba from water was also reported using paired-ion electrospray ionization (ESI) mass spectrometry [14]. Furthermore, several MS-based dicamba analytical methods have been reported for food items [17,18], water and agricultural cleanouts [13,14,16,19,20,21,22], air samplers [6,23,24], crops like apple, pepper, brown rice, and soybean [25], tobacco [26], barley [27], soybean [28], rice [29], as well as in other agricultural commodities [30]. Most of these protocols rely on triple quadrupole and/or higher resolution MS instruments, although quantification procedures using LC–diode array detection (DAD) [24] and single-quadrupole LC–MS [6,31] have been reported.

Single-quadrupole MS analyzers offer low mass resolution, are accurate in their application, and are known to be very robust and simple to use [32]. With selected ion monitoring (SIM), sufficient analyte sensitivity can often be gained with single-quadrupole MS analyzers [32], thus making it a good choice for single compound analysis, identification, and quantitation. Based on sample complexity, matrix effects are commonly observed in any analytical MS method. Since the matrix effects originate from the ionization source, the SIM functionality is also susceptible to matrix interferences especially in the case of complex matrices, which can be addressed through introducing an internal standard (IS) in the sample preparation workflow. Use of an isotopically labeled analog of the target analyte as an IS is the most effective way to correct for matrix effects, since, theoretically, the same degree of ion suppression or enhancement is observed for the target analyte and its isotopically labeled analog [33,34,35]. Moreover, using an IS also rectifies any variation other than that related to the amount of the analyte present in a sample, such as variability in dilution, evaporation, degradation, recovery, adsorption, derivatization, injection, and detection [36]. An isotopically labeled or structurally similar analog of the target analyte is used as an IS, and a known amount of IS is introduced in a sample at the very beginning of the sample preparation, allowing simultaneous steps followed throughout the analysis. However, the analytical method must be able to distinguish between the target analyte and the IS by either retention time or molecular mass. Therefore, IS-based quantification is a good choice for analysis and a commonly used approach in GC–MS- and LC–MS-based analytical methodologies [33,34,35,36,37,38,39]. In this study, we report a detailed evaluation of a single-quadrupole LC–MS method with SIM mode detection and use of an isotopically labeled IS and relative response factor (RRF)-based direct quantification to monitor dicamba in air and water samples. The evaluated method offers an economical and robust option for rapid, efficient, and high-throughput detection of dicamba.

## 2. Materials and Methods

### 2.1. Chemicals and Reagents

A reference standard of dicamba (3,6-dichloro-2-methoxybenzoic acid; catalog number 45430) and the IS, D_3_-dicamba (3,6-dichloro-2-(methoxy-d_3_) benzoic acid; catalog number 34233), were purchased from Sigma-Aldrich (St. Louis, MO, USA). Acetonitrile (ACN), Optima™ LC–MS Grade with 0.1% formic acid (*v*/*v*) (catalog number LS120-4), and methanol, Optima™ LC–MS Grade (catalog number A456-4), were purchased from Fisher Scientific (Pittsburgh, PA, USA). The commercial dicamba formulation used in the study, XtendiMax^®^ with VaporGrip^®^ Technology (diglycolamine salt of dicamba; Bayer Crop Science, Research Triangle Park, NC, USA), was sourced locally. The XAD^®^-2 sorbent and polyurethane foam (PUF) plug in air samplers was purchased from SKC, Inc. (Eighty Four, PA, USA). 

### 2.2. LC–MS Analysis

**Figure 2:**
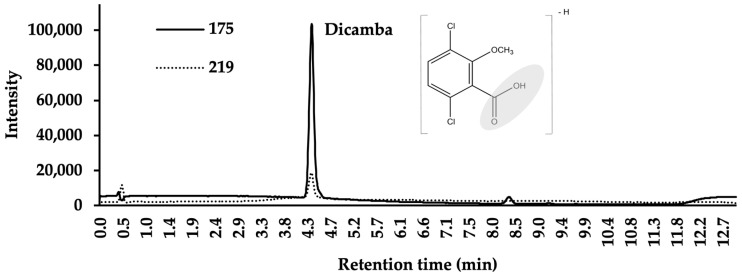
Detection of dicamba in selected ion monitoring (SIM) mode with *m*/*z* 219 (parent ion, dashed line) and *m*/*z* 175 (fragment ion, solid line) ions shown (1 µg mL^−1^). Shaded region of dicamba structure shows COOH fragment that is lost from precursor ion (*m*/*z* 219) that results in fragment ion (*m*/*z* 175).

A routine LC–MS method was established for the efficient separation and identification of dicamba. LC–MS analysis was performed using an Agilent 6135 single-quadrupole mass spectrometer equipped with Jet Stream technology electrospray ionization (ESI) source, and the MS was coupled to a 1290 Infinity II UHPLC system with a diode array detector (Agilent Technologies, Santa Clara, CA, USA). The chromatographic separation of dicamba was achieved on an EclipsePlusC18 RRHD column (1.8 µm, 2.1 × 50 mm; Agilent) connected to a Zorbax SB-C18 analytical guard column (5 µm, 4.6 × 12.5 mm; Agilent). Water (A) and ACN (B), both with 0.1% formic acid (*v*/*v*), were used as mobile phase solvents at a flow rate of 0.3 mL min^−1^. The LC solvent gradient program was set as described in Table 1 with a total runtime of 13 min. Column temperature maintained at 30 °C, a pure analytical grade dicamba standard was found to elute at 4.4 min (Figure 2). The Jet Stream ESI source was operated in negative-ion SIM mode with nozzle and capillary voltages set at 2000 V and 4000 V, respectively. Sheath gas temperature was set at 360 °C with a flow of 13 mL min^−1^ and drying gas temperature kept at 350 °C with a flow of 12 mL min^−1^. Data analyses were performed using OpenLAB CDS ChemStation software version C.01.08[210] (Agilent Technologies, Santa Clara, CA, USA). 

### 2.3. Dicamba Quantification

Quantification was performed by the following rearrangement of the RRF equation:

(1) $$RRF=\left(\frac{Peak\;area\;A}{Conc.A}\right)\times \left(\frac{Conc.IS}{Peak\;area\;IS}\right)$$

In order to solve for Concentration A (“Conc. A”) similar to that previously reported for the direct quantification of compounds [39] as follows:

(2) $$Conc.\;A=Peak\;area\;A\times \left(\frac{Conc.IS}{Peak\;area\;IS}\right)\times \left(\frac{1}{RRF}\;\right)$$

where A is the analyte, IS the internal standard, and RRF is the relative response factor. The response factor (RF) values for the analyte and the IS were obtained by division of peak area by the concentration, and RRF was calculated based on the division of RF of an analyte to the RF of the IS. The values including peak areas of the analyte and IS, the concentration of IS, and RRF were then used to calculate the analyte concentration in the samples. To further minimize any smaller variations in the RRF, the average value of RRF was obtained by analyzing five serially diluted concentrations of dicamba (10, 5, 2.5, 1.25, and 0.625 μg mL^−1^) mixed with the IS, D_3_-dicamba, which was achieved by mixing 190 μL from each concentration of dicamba with 10 μL of 100 μg mL^−1^ of the D_3_-dicamba solution. 

## 3. Results and Discussion

### 3.1. SIM Ion Selection and Optimization

**Figure 3:**
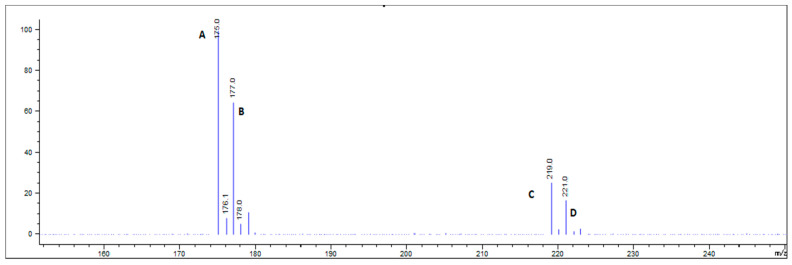
Mass spectrometry (MS) full scan spectrum of dicamba analytical standard showing isotopic pattern corresponding to ^35^Cl *m*/*z* 175 (A) and *m*/z 219 (C), and ^37^Cl *m*/*z* 177 (B) and *m*/*z* 221 (D).

The MS analysis was performed using the SIM function to achieve maximum sensitivity [32]. The SIM function in single-quadrupole MS allows detection of a specific mass-to-charge ratio (*m*/*z)* and to collect the data at that *m*/*z* of interest rather than stepping the MS over an extensive range of masses, thus achieving better detection sensitivity. In this method, the selection of all SIM ions for dicamba and D_3_-dicamba was made upon full scan analyses of pure analytical standards, which showed consistent patterns of the fragment ions and their intensities. Dicamba in ESI negative mode showed the *m*/*z* 219 [M − H]^−^ (Figure 3), which is its commonly observed parent ion. Indeed, this ion was previously reported to be monitored using SIM mode with single-quadrupole MS by others measuring dicamba in air samples collected from humidomes [6]. While performing the full scan analysis of dicamba, however, we also found the fragment *m*/*z* 175 [M-COOH]^−^ corresponding to the same chromatographic peak of dicamba (Figure 3). The *m*/*z* 175 ion is also a commonly found fragment ion of dicamba reported in several studies as the MS2 product ion within MS/MS experiments [12,13,17,40]. In our analysis, the intensity of *m*/*z* 175 was observed to be significantly higher than the intensity of *m*/*z* 219 (Figure 2); therefore, both *m*/*z* 175 and 219 were included as monitored SIM ions in the method. Not only does monitoring the *m*/*z* 175 ion provide increased sensitivity, but it also provides additional confirmation to the identity of dicamba. Accordingly, the *m*/*z* 178 and 222 ions were found to be the optimal SIM ions for the IS, D_3_-dicamba (data not shown).

One thing to note about selecting SIM ions in single-quadrupole MS-based detection of dicamba is that the molecule contains chlorine atoms covalently linked to the C3 and C6 positions of the benzenoid ring (Figure 1). Chlorine has two stable isotopes found in nature, ^35^Cl and ^37^Cl, which occur at an isotopic abundance ratio of 3:1 ^35^Cl:^37^Cl. This relative isotopic abundance of chlorine is well known in mass spectrometry and is commonly used for positive identification of chlorinated compounds. When molecules have two chlorine atoms, an expected isotopic ratio of 3:2 for M:M+2 is typically observed. Indeed, in our full scans of ions of the dicamba standard, we observed *m*/*z* 219 and 221, as well as the corresponding fragment ions, *m*/*z* 175 and 177, in 3:2 ratios (Figure 3). In MS/MS, both sets of ion pairs are typically monitored during dicamba analysis. In a previous study by Mueller et al. [31], the *m*/*z* 177 and 180 ions were monitored in SIM mode for single-quadrupole MS-based detection of dicamba and D_3_-dicamba, respectively. Because these likely correspond to the isotopically less abundant (^37^Cl) fragment ions, we recommend monitoring the *m*/*z* 175 and 178 fragment ions for dicamba and D_3_-dicamba. Ultimately, the total peak areas of both the more isotopically abundant (^35^Cl) precursor and fragment ions for the analyte (*m*/*z* 175 + 219) and for the IS (*m*/*z* 178 + 222) were subsequently used in the calculations.

We also optimized the fragmentor voltage to achieve increased sensitivity and selectivity for the selected SIM ions. The fragmentor in the Agilent 6135 single-quadrupole mass spectrometer system is located at the exit end of the capillary and voltage applied to it affects ion transmission as well as causes fragmentation. In general, the higher the fragmentor voltage, the more fragmentation will occur. Fragmentor voltage gives the ions a “push” that helps them traverse the relatively high-pressure region between the exit of the capillary and skimmer. The fragmentor voltage here is not to be confused with fragmentor voltage commonly reported with triple-quadrupole instruments, which is applied to its collision cell. The optimum voltage was obtained by analyzing 1 µg mL^−1^ dicamba standard at different voltage values ranging from 0 to 120 V, which showed the highest response at 60 V (Table S1, Supplementary Materials) and was thus selected as the optimum value for the method.

### 3.2. Method Performance

The performance of the analytical method including sample preparation and instrumental method is tested in general analytical method validation studies [41]. In the present study, the method performance was evaluated considering the following validation parameters.

#### 3.2.1. Linearity

The linearity of the method was determined by obtaining the linear regression of the calibration curve with seven serially diluted dicamba standards (24.4–1562.5 ng mL^−1^) prepared in pure solvent and plotting the peak area against the concentration of corresponding calibration standards. The analysis was performed in triplicate on three different days. All calibration points were within the acceptance range and showed relative standard deviation (RSD) ± 20 with a coefficient of determination (R2) ≥ 0.990 (Table S2, Supplementary Materials).

#### 3.2.2. Sensitivity

The instrumental method sensitivity was determined by the limit of detection (LOD) and limit of quantification (LOQ). The LOD was determined based on the observed signal-to-noise ratio (S/N) of 3, whereas the LOQ was determined based on the observed S/N of 10. Using these S/N ratios, the LOD and LOQ of the instrument itself were observed to be 9 and 24 ng mL^–1^, respectively. Once considering the concentration factors from the dicamba extraction procedures detailed below from water and air samples, LODs of 0.1 ng mL^−1^ and 1 ng mL^−1^, respectively, were obtained.

#### 3.2.3. Recovery and Repeatability

Method recovery experiments were carried out by external standard addition of dicamba standards at three different concentration levels on three different days for both water and air sample types. Quantification was done by using calculations described in Section 2.3. 

To mimic extraction from air samples, dicamba standard was added at 1 (Level 1), 5 (Level 2), and 10 (Level 3) ng mL^−1^ in a 40 mL volume of methanol in the sample preparation step. The IS, D_3_-dicamba, was added similarly at the concentrations of 2.5 (Level 1), 6.5 (Level 2), and 11.25 (Level 3) ng mL^−1^. The addition was made directly on XAD^®^-2 sorbent resins 16 gm, which was placed in 50 mL centrifuge tubes. The tubes were kept at room temperature for 4 h and extracted as described in Section 3.3., and the residues were dissolved in 500 µL solvent. The average recoveries for air samples for three different fortified concentrations were calculated to be within the range of 88–124% (Table S3, Supplementary Materials; Table 2). 

To mimic extraction from water samples, dicamba standard was added at 0.1 (Level 1), 1 (Level 2), and 10 (Level 3) ng mL^−1^ in 500 mL of sample volume. The IS, D_3_-dicamba, was also added similarly at concentrations of 0.2 (Level 1), 1 (Level 2), and 10 (Level 3) ng mL^−1^. Samples were then kept at room temperature for 4 h, followed by the extraction procedure as mentioned in Section 3.4. The final residues were dissolved in 500 µL solvent. The average recoveries for water samples for three different fortified concentrations were found within the range of 106–128% (Table S4, Supplementary Materials; Table 2). 

#### 3.2.4. Matrix Effect Evaluation

The evaluation of matrix effects was performed by analyzing dicamba standards prepared in pure solvent, sorbent resin+PUF matrix solvent, and the water sample matrix solvent. A percent matrix effect less than 100% suggests ion suppression, whereas an effect greater than 100% indicates ion enhancement. Little effect was observed for water (within 93–105%), which was expected for tap water. At the same time, a higher matrix effect was observed for sorbent resin+PUF, between 35% and 46% (Table S5, Supplementary Materials), which likely originates from materials used in the manufacturing of the sorbent resins and PUF. Nonetheless, the recoveries reported in Section 3.2.3. suggest that the isotopically labeled IS with RRF-based analysis sufficiently accounts for the matrix interference to attain accurate quantification. 

### 3.3. Air Sample Analysis

**Figure 4:**
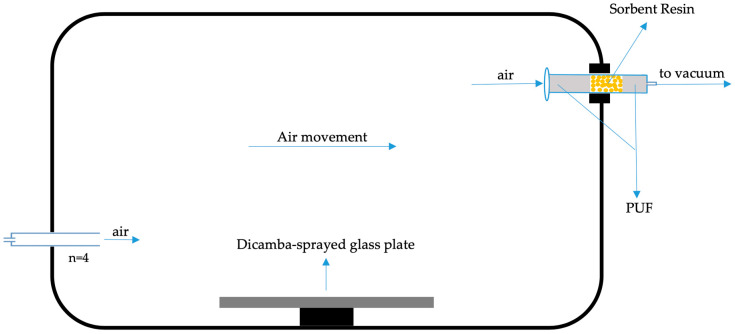
Schematic diagram of the vapor chamber (side view) used in the experiment.

To fully evaluate air sample extraction, we performed vapor chamber experiments using glass plates (Figure 4) similar to those conducted with plants in other herbicide volatility studies [6,23]. Glass plates with a surface area of 190 cm^2^ were sprayed with selected dicamba formulation using a single-nozzle, track-mounted research sprayer calibrated to deliver 140 L ha^−1^ per pass using a Turbo Teejet Induction nozzle (TTI 11002) (Teejet Technologies, Springfield, IL, USA). The dicamba diglycolamine (DGA) salt formulation of dicamba (XtendiMax^®^) was applied at rates of 560 and 2240 g ae ha^−1^. After spraying, glass plates were transferred to the vapor chambers. The vapor chambers had an internal volume of 6.2 L and holes at both ends to maintain air movement inside the chamber and across the treated surface. Four holes (diameter 0.794 cm) for the air inlet and a single hole (1.27 cm) for the air outlet were made in the opposite direction of the chamber. Airflow rate was maintained at 310 mL min^−1^ with Dwyer airflow meters (Dwyer Instrument, Michigan City, IN, USA), resulting in a full turnover of air in the chamber every 20 min. The chambers were placed in a controlled environment growth chamber set to 35 °C and 40% relative humidity during the treatment period. Air samplers for the collection of dicamba were connected to chambers using glass tubes containing 270 mg of XAD-2 sorbent. Both ends of the glass tubes were sealed with 1 and 0.5 cm PUF plugs to avoid any spill or movement of polymer sorbent particles inside the tube. Air sampling was conducted for a period of 48 h. The XAD-2 sorbent and PUFs were carefully transferred into 15 mL polypropylene centrifuge tubes and 10 mL methanol was added. Next, 25 µL of 100 µg mL^−1^ D_3_-dicamba solution were also added and tubes were kept for overnight shaking at 4 °C to elute dicamba from the sorbent and from the PUF into the solvent. The next day, the methanol was transferred to new tubes and concentrated to dryness using the TurboVap^®^ LV system (Biotage LLC, Charlotte, NC, USA) under N_2_ gas at 30 °C. Dicamba residues were then resuspended to a final volume of 200 μL in ACN with 0.1% (*v*/*v*) formic acid and 20 µL were analyzed using the above described single-quadrupole LC–MS method. The results reproducibly show an increased amount of dicamba detected when comparing application rates of 560 and 2240 g ae ha^−1^. These data indicate that a stable drift and/or volatilization of dicamba occurred off of the glass plates (Table 3). It is important to note that this non-reactive surface does not necessarily reflect a plant surface where dicamba interacts with surface ions and is absorbed into the leaves over time. These data do show, however, the precision and reproducibility of the developed method for extracting dicamba from PUF plugs and of the subsequent analysis using our established single-quadrupole LC–MS method.

### 3.4. Water Sample Analysis 

Previously reported dicamba contaminations detected in agricultural sprayer hose cleanouts following dicamba application at 560 g ae ha^−1^ (3000 µg mL^−1^ by volume) were found to be up to 16 µg mL^−1^ in 392 mL of sequestered solution [20]. As dicamba injury can occur to susceptible crops at 0.01% of the use rate [42] (i.e., 300 ng mL^−1^), we evaluated our dicamba water extraction protocol and LC–MS detection method near this level. Following an XtendiMax^®^ (dicamba DGA salt) application using a four-nozzle hand boom at 560 g ae ha^−1^ in tap water, three 500 mL tap water rinses were passed through the boom hose and collected in four 125 mL fractions (one from each nozzle). Assuming our initial rinse outs were likely to be near 16 µg mL^−1^ dicamba per 500 mL like those reported by others [20], we diluted our rinses 50-fold (to reach approximately 300 ng mL^−1^) as follows. From each of the four 125 mL samples for each rinse, a 10 mL aliquot (i.e., a 50× dilution from the original 500 mL) was diluted into 500 mL tap water adjusted to pH 2 with 6M HCl and processed per a previously described protocol [12] with minor modifications. For samples obtained from Rinse 1, 200 µL of 100 µg mL^−1^ D_3_-dicamba IS were added, whereas for those from Rinse 2 and Rinse 3, 100 µL of 100 µg mL^−1^ D_3_-dicamba were added. The subsequent extraction was performed using solid-phase extraction (SPE) with ISOLUTE ENV+ cartridges (200 mg, 6 mL, Biotage). The cartridges were conditioned by passing 5 mL each of methanol and non-sample water at pH 2. After conditioning, the prepared 500 mL samples were passed through the cartridges followed by cleaning with 5 mL non-sample pH 2 water. Cartridges were then dried by keeping them on the vacuum manifold for 2–3 min, and then eluted with 8 mL mixture of acetone:ethyl acetate (1:1). The elutant was collected in 10 mL glass tubes, and any leftover fraction of water was removed by using a pasture pipette. The organic solvent was then evaporated to dryness at 30 °C under N_2_ using a TurboVap^®^ LV system (Biotage). Dicamba residue was reconstituted in 500 µL of ACN with 0.1% FA and 5 µL subsequently analyzed using the above described LC–MS method. 

Measured concentrations in water samples following SPE extraction and LC–MS detection were highly reproducible across replicates from all samples (Table 4). Indeed, the 50× dilution of our initial rinse was found to be near 300 ng mL^−1^ (Table 4), thus confirming previous observations that initial hose cleanouts approach 16 µg mL^−1^ [20]. Considering both the instrument LOD of 9 ng mL^−1^, based on the observed S/N of 3, and the concentration factor from the SPE extraction procedure, our method LOD was determined to be 0.1 ng mL^−1^. This is similar to that reported by Mueller and Steckel [6] and is significantly lower than that reported by Hu et al., who used LC atmospheric pressure chemical ionization MS (LC–APCI–MS) and found an LOD of 500 ng mL^−1^ [19]. While previous studies using LC–MS/MS found method LODs ranging from 0.003 to 0.01 ng mL^−1^ [12,13], the SPE and single-quadrupole LC–MS method described here provides sufficient sensitivity by orders of magnitude to detect dicamba residue in agricultural hose cleanouts [20].

## 4. Conclusions

The evaluation of the reported IS-based single-quadrupole LC–MS method using an RRF-based direct quantification approach suggests that it is robust and sufficiently sensitive for monitoring dicamba in agriculturally relevant air and water samples. The method relies on SIM mode to establish sensitivity, shows LOQs of 0.1 and 5 ng mL^−1^, and LODs of 0.1 and 1 ng mL^−1^, for dicamba in water and air, respectively, and has acceptable reproducibility and repeatability. Detection limits reported by our evaluated method are within the range of previously reported LODs, which are 0.003–5 ng mL^−1^ using tandem MS [12,13,26,30,40]. The selection of a combination of precursor and fragment ions in SIM mode was found to improve detection sensitivity, and provides additional positive identification of dicamba. The higher intensity of the dicamba fragment ion (*m*/*z* 175) than its precursor ion (*m*/*z* 219; Figure 2) reflects the fragmentation behavior of the molecule in the ionization source, hence highlighting the need for in-house instrumental optimization when it comes to selecting SIM ions to monitor by single-quadrupole LC–MS. Despite the high ion suppression observed due to the matrix effect in the sorbent resin+PUF matrix (Table S5), recoveries were still acceptable (Table 2), thus underscoring the importance of D_3_-dicamba as an IS and the use of RRF-based analysis for dicamba quantification. 

## Figures and Tables

**Table 1:** Liquid chromatography (LC) solvent gradient program.

Time	A (%)	B (%)	Flow µL min^−1^
0	80	20	300
2	80	20	300
10	25	75	300
11	25	75	300
12	80	20	300
13	80	20	300

**Table 2:** Method recovery in air and water samples. RSD, relative standard deviation.

		Concentration of Dicamba Added (ng mL^−1^)	% Recovery	RSD %
**Water**	Level 1	0.1	128	10.3
	Level 2	1.0	108	5.6
	Level 3	10.0	110	4.9
**Air**	Level 1	1.0	124	9.2
	Level 2	5.0	88	5.5
	Level 3	10.0	88	3.3

**Table 3:** Dicamba levels in air samples.

Dicamba Application (g ae ha^−1^)	Total Amount of Dicamba (ng) Per Sample					
	Rep 1	Rep 2	Rep 3	Rep 4	Average	RSD %
560	729.7	763.2	549.4	741.1	668.7	14.8
2240	5172.8	4592.6	3975.1	4480.7	4092.2	12.0

**Table 4:** Dicamba quantification from 500 mL water samples. Conc., concentration.

	(ng mL^−1^)					
Sample	Rep 1	Rep 2	Rep 3	Rep 4	Average Conc.	RSD %
**Rinse 1**	296.5	278.0	293.7	274.1	285.6	4
**Rinse 2**	115.1	111.9	114.1	109.5	112.7	2
**Rinse 3**	76.8	72.1	70.6	76.3	74.0	4

## Supplementary Materials

The following are available online, Table S1: Fragmentation voltage optimization for MS; Table S2: Linearity of the dicamba standards; Table S3: Dicamba recovery in air samples; Table S4: Dicamba recovery in water samples; Table S5: Matrix effect evaluation.

## References

[1] Gleason C., Foley R.C., Singh K.B. (2011). Mutant analysis in Arabidopsis provides insight into the molecular mode of action of the auxinic herbicide dicamba. PLoS ONE.

[2] Riter L.S., Sall E.D., Pai N., Beachum C.E., Orr T.B. (2020). Quantifying Dicamba Volatility under Field Conditions: Part, I., Methodology. J. Agric. Food Chem..

[3] Egan J.F., Mortensen D.A. (2012). Quantifying vapor drift of dicamba herbicides applied to soybean. Env. Toxicol. Chem..

[4] Sall E.D., Huang K., Pai N., Schapaugh A.W., Honegger J.L., Orr T.B., Riter L.S. (2020). Quantifying Dicamba Volatility under Field Conditions: Part II, Comparative Analysis of 23 Dicamba Volatility Field Trials. J. Agric. Food Chem..

[5] Mueller T.C. (2015). Methods To Measure Herbicide Volatility. Weed Sci..

[6] Mueller T.C., Steckel L.E. (2019). Dicamba volatility in humidomes as affected by temperature and herbicide treatment. Weed Technol..

[7] Strachan S.D., Casini M.S., Heldreth K.M., Scocas J.A., Nissen S.J., Bukun B., Lindenmayer R.B., Shaner D.L., Westra P., Brunk G. (2010). Vapor Movement of Synthetic Auxin Herbicides: Aminocyclopyrachlor, Aminocyclopyrachlor-Methyl Ester, Dicamba, and Aminopyralid. Weed Sci..

[8] Behrens R., Lueschen W.E. (1979). Dicamba Voiatility. Weed Sci..

[9] Willett C.D., Grantz E.M., Lee J.A., Thompson M.N., Norsworthy J.K. (2019). Soybean response to dicamba in irrigation water under controlled environmental conditions. Weed Sci..

[10] Picó Y., Blasco C., Font G. (2004). Environmental and food applications of LC-tandem mass spectrometry in pesticide-residue analysis: An overview. Mass Spectrom. Rev..

[11] Malik A.K., Blasco C., Picó Y. (2010). Liquid chromatography-mass spectrometry in food safety. J. Chromatogr. A.

[12] Majzik E.S., Tóth F., Benke L., Kiss Z. (2006). SPE-LC-MS-MS determination of phenoxy acid herbicides in surface and ground water. Chromatographia.

[13] McManus S.-L., Moloney M., Richards K., Coxon C., Danaher M. (2014). Determination and Occurrence of Phenoxyacetic Acid Herbicides and Their Transformation Products in Groundwater Using Ultra High Performance Liquid Chromatography Coupled to Tandem Mass Spectrometry. Molecules.

[14] Xu C., Armstrong D.W. (2013). High-performance liquid chromatography with paired ion electrospray ionization (PIESI) tandem mass spectrometry for the highly sensitive determination of acidic pesticides in water. Anal. Chim. Acta.

[15] Catalina M.I., Dallüge J., Vreuls R.J.J., Brinkman U.A.T. (2000). Determination of chlorophenoxy acid herbicides in water by in situ esterification followed by in-vial liquid-liquid extraction combined with large-volume on-column injection and gas chromatography-mass spectrometry. J. Chromatogr. A.

[16] Rodríguez I., Rubí E., González R., Quintana J.B., Cela R. (2005). On-fibre silylation following solid-phase microextraction for the determination of acidic herbicides in water samples by gas chromatography. Anal. Chim. Acta.

[17] Sack C., Vonderbrink J., Smoker M., Smith R.E. (2015). Determination of Acid Herbicides Using Modified QuEChERS with Fast Switching ESI+/ESI- LC-MS/MS. J. Agric. Food Chem..

[18] Steinborn A., Alder L., Spitzke M., Dörk D., Anastassiades M. (2017). Development of a QuEChERS-Based Method for the Simultaneous Determination of Acidic Pesticides, Their Esters, and Conjugates Following Alkaline Hydrolysis. J. Agric. Food Chem..

[19] Hu J.-Y., Aizawa T., Magara Y. (1999). Analysis of pesticides in water with liquid chromatography/atmospheric pressure chemical ionization mass spectrometry. Water Res..

[20] Cundiff G.T., Reynolds D.B., Mueller T.C. (2017). Evaluation of Dicamba Persistence among Various Agricultural Hose Types and Cleanout Procedures Using Soybean (Glycine max) as a Bio-Indicator. Weed Sci..

[21] Osborne P.P., Xu Z., Swanson K.D., Walker T., Farmer D.K. (2015). Dicamba and 2,4-D residues following applicator cleanout: A potential point source to the environment and worker exposure. J. Air Waste Manag. Assoc..

[22] Quintana J.B., Rodil R., Muniategui-Lorenzo S., López-Mahía P., Prada-Rodríguez D. (2007). Multiresidue analysis of acidic and polar organic contaminants in water samples by stir-bar sorptive extraction-liquid desorption-gas chromatography-mass spectrometry. J. Chromatogr. A.

[23] Ouse D.G., Gifford J.M., Schleier J., Simpson D.D., Tank H.H., Jennings C.J., Annangudi S.P., Valverde-Garcia P., Masters R.A. (2018). A New Approach to Quantify Herbicide Volatility. Weed Technol..

[24] Bish M.D., Farrell S.T., Lerch R.N., Bradley K.W. (2019). Dicamba Losses to Air after Applications to Soybean under Stable and Nonstable Atmospheric Conditions. J. Environ. Qual..

[25] Shin E.H., Choi J.H., Abd El-Aty A.M., Khay S., Kim S.J., Im M.H., Kwon C.H., Shim J.H. (2011). Simultaneous determination of three acidic herbicide residues in food crops using HPLC and confirmation via LC-MS/MS. Biomed. Chromatogr..

[26] Liu S., Bian Z., Yang F., Li Z., Fan Z., Zhang H., Wang Y., Zhang Y., Tang G. (2015). Determination of multiresidues of three acid herbicides in tobacco by liquid chromatography/tandem mass spectrometry. J. AOAC Int..

[27] Díez C., Traag W.A., Zommer P., Marinero P., Atienza J. (2006). Comparison of an acetonitrile extraction/partitioning and “dispersive solid-phase extraction” method with classical multi-residue methods for the extraction of herbicide residues in barley samples. J. Chromatogr. A.

[28] Hua K., Xiao-Gang C., Yu-Xia H., Chuan-Lai X. (2006). Simultaneous determination of 13 phenoxy acid herbicide residues in soybean by GC-ECD. Anal. Lett..

[29] Koesukwiwat U., Sanguankaew K., Leepipatpiboon N. (2008). Rapid determination of phenoxy acid residues in rice by modified QuEChERS extraction and liquid chromatography-tandem mass spectrometry. Anal. Chim. Acta.

[30] Guo H., Riter L.S., Wujcik C.E., Armstrong D.W. (2016). Quantitative analysis of dicamba residues in raw agricultural commodities with the use of ion-pairing reagents in LC-ESI-MS/MS. Talanta.

[31] Mueller T.C., Wright D.R., Remund K.M. (2013). Effect of Formulation and Application Time of Day on Detecting Dicamba in the Air under Field Conditions. Weed Sci..

[32] Dunn W.B., Jameson D., Verma M., Westerhoff H.V. (2011). Mass spectrometry in systems biology:An introduction. Methods in Systems Biology.

[33] Colby B.N., McCaman M.W. (1979). A comparison of calculation procedures for isotope dilution determinations using gas chromatography mass spectrometry. Biol. Mass Spectrom..

[34] Berg T., Strand D.H. (2011). 13C labelled internal standards-A solution to minimize ion suppression effects in liquid chromatography-tandem mass spectrometry analyses of drugs in biological samples?. J. Chromatogr. A.

[35] Panuwet P., Hunter R.E., D’Souza P.E., Chen X., Radford S.A., Cohen J.R., Marder M.E., Kartavenka K., Ryan P.B., Barr D.B. (2016). Biological Matrix Effects in Quantitative Tandem Mass Spectrometry-Based Analytical Methods: Advancing Biomonitoring. Critical Reviews in Analytical Chemistry.

[36] Tan A., Lévesque I.A., Lévesque I.M., Boudreau N., Lévesque A. (2011). Analyte and internal standard cross signal contributions and their impact on quantitation in LC-MS based bioanalysis. J. Chromatogr. B.

[37] Chakravarthy V.K., Babu G.K., Dasu R.L., Prathyusha P., Kiran G.A. (2011). The role of relative response factor in related substances method developement by high performace liquid chromatography (HPLC). Rasayan J. Chem..

[38] Nilsson L.B., Eklund G. (2007). Direct quantification in bioanalytical LC-MS/MS using internal calibration via analyte/stable isotope ratio. J. Pharm. Biomed. Anal..

[39] Rome K., Mcintyre A. (2012). Intelligent use of relative response factors in gas chromatography-flame ionisation detection. Chromatogr. Today.

[40] Xiong W., Tao X., Pang S., Yang X., Tang G.L., Bian Z. (2014). Separation and quantitation of three acidic herbicide residues in tobacco and soil by dispersive solid-phase extraction and UPLC-MS/MS. J. Chromatogr. Sci..

[41] Thompson M., Ellison S.L.R., Wood R. (2002). Harmonized guidelines for single-laboratory validation of methods of analysis (IUPAC Technical Report). Pure Appl. Chem..

[42] Kelley K.B., Riechers D.E. (2003). Gene expression analysis of auxinic herbicide injury in soybeans (Abstract). Weed Sci. Soc. Am..

